# Abnormal Profiles of Local Functional Connectivity Proximal to Focal Cortical Dysplasias

**DOI:** 10.1371/journal.pone.0166022

**Published:** 2016-11-18

**Authors:** René M. H. Besseling, Jacobus F. A. Jansen, Anton J. A. de Louw, Mariëlle C. G. Vlooswijk, M. Christianne Hoeberigs, Albert P. Aldenkamp, Walter H. Backes, Paul A. M. Hofman

**Affiliations:** 1 Epilepsy center Kempenhaeghe, Heeze, the Netherlands; 2 Research School for Mental Health & Neuroscience, Maastricht University, Maastricht, the Netherlands; 3 Department of Radiology, Maastricht University Medical Center, Maastricht, the Netherlands; 4 Department of Neurology, Maastricht University Medical Center, Maastricht, the Netherlands; Hospital for Sick Children, CANADA

## Abstract

**Introduction:**

Focal cortical dysplasia (FCD) is a congenital malformation of cortical development that often leads to medically refractory epilepsy. Focal resection can be an effective treatment, but is challenging as the surgically relevant abnormality may exceed the MR-visible lesion. The aim of the current study is to develop methodology to characterize the profile of functional connectivity around FCDs using resting-state functional MRI and in the individual patient. The detection of aberrant connectivity may provide a means to more completely delineate the clinically relevant lesion.

**Materials and Methods:**

Fifteen FCD patients (age, mean±SD: 31±11 years; 11 males) and 16 matched healthy controls (35±9 years; 7 males) underwent structural and functional imaging at 3 Tesla. The cortical surface was reconstructed from the T1-weighted scan and the registered functional MRI data was spatially normalized to a common anatomical standard space employing the gyral pattern. Seed-based functional connectivity was determined in all subjects for all dysplasia locations. A single patient was excluded based on an aberrant FCD seed time series. Functional connectivity as a function of geodesic distance (along the cortical surface) was compared between the individual patients and the homotopic normative connectivity profiles derived from the controls.

**Results:**

In 12/14 patients, aberrant profiles of functional connectivity were found, which demonstrated both hyper- and hypoconnectivity as well as combinations. Abnormal functional connectivity was typically found (also) beyond the lesion visible on structural MRI, while functional connectivity profiles not related to a lesion appeared normal in patients.

**Conclusion:**

This novel functional MRI technique has potential for delineating functionally aberrant from normal cortex beyond the structural lesion in FCD, which remains to be confirmed in future research.

## Introduction

Focal cortical dysplasias (FCDs) are congenital malformations of cortical development that are highly epileptogenic, and often give rise to medically refractory epilepsy [1,2]. Resection of the dysplasia can be an effective treatment, and FCD is the most common etiology in children and the third most frequent finding in adults undergoing epilepsy neurosurgery [3].

Accurate visualization through neuroimaging is crucial for surgical planning and outcome. Histopathological examination of surgical specimens has demonstrated that 80% of patients who had a complete resection become seizure free, compared to only 20% for incomplete resections [3]. Advances in MR technology such as higher field strengths and improved head coils have significantly increased the diagnostic yield, and allow the detection and demarcation of smaller and more subtle lesions [4].

Semi-automatic approaches that enhance morphological characteristics of FCD such as cortical thickening and blurring of the gray matter- white matter interface may aid lesion detection [5–7]. The presence of underlying white matter abnormalities may also help to more completely delineate the region of aberrant cortex. For example, it has recently been reported that postsurgical outcome is significantly better in patients with an FCD-typical white matter transmantle sign compared to those without [8].

In an attempt to improve sensitivity for white matter abnormalities compared to conventional structural imaging, diffusion weighted imaging (DWI) has been used to study white matter microstructure and structural connectivity. Compared to the contralateral hemisphere, lower fractional anisotropy (FA) was found in directly adjacent white matter, indicative of reduced microstructural tissue integrity, as well as reduced volume of nearby major white matter tracts [9,10]. Also distal effects of reduced FA have been found, in major white matter tracts projecting to or from the FCD [11].

Alternatively, the functional connectivity of FCDs may be investigated to improve the characterization of aberrant tissue. Since functional connectivity analysis assesses similarities in gray matter time series, this may provide a more direct way to demarcate aberrant cortex. Furthermore, since functional and structural connectivity are under mutual influence, functional remodeling is to be expected in relation to the already described (micro)structural changes [8,9,11,12].

In this study, we describe methodology to study functional connectivity as a function of distance from the MR-visible lesion in individual FCD patients compared to homotopic connectivity profiles derived from a group of healthy controls. Distance is defined as the geodesic distance (along the cortical surface), as an approximation to the distance of local information flow along juxtacortical short association fibers. The aim is to detect local abnormalities in functional connectivity, potentially reflecting the broader abnormality of the underlying tissue.

## Materials and Methods

### Ethics statement

Written informed consent was obtained from all participants and the study was approved by the review boards of both Maastricht University Medical Center and epilepsy center Kempenhaeghe.

### Subjects

**Fig 1:**
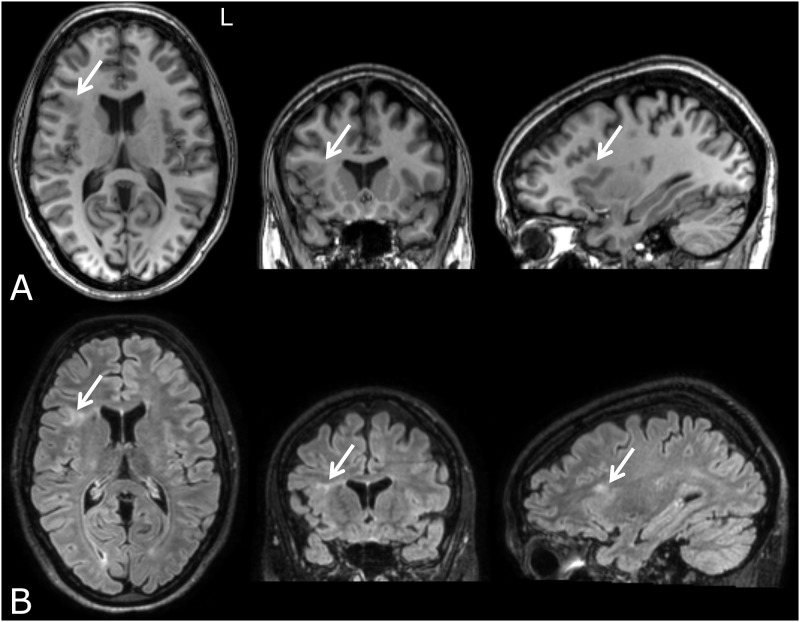
Conventional structural MRI features of focal cortical dysplasia in a representative subject (patient 1) for 3 orthogonal slices. The abnormalities are indicates by arrows; in the T1-weighted images (A) these are cortical thickening and blurring of the gray matter-white matter interface; in the FLAIR-weighted images (B), hyperintensities in the underlying white matter can be observed.

Fifteen clinical patients with FCD-related epilepsy were recruited at our specialized epilepsy referral center (age, mean±SD: 31±11 years; 11 males), as well as 16 healthy controls (35±9 years; 7 males). The controls were matched for sex, age and education level. The diagnosis was based on concordance between seizure semiology, EEG findings, and neuroimaging [1,2,13]. Briefly, this involved recurrent stereotyped seizures and focal interictal and/or ictal EEG abnormalities that coincided with an FCD-concordant lesion on MRI [14]. Relevant imaging features included, among others, abnormal gyral pattern, increased cortical thickness, transmantle sign, and blurring of the gray matter-white matter interface [2,7,15]; for an example, see Fig 1. Information on individual dysplasia locations and EEG findings are given in Table 1. None of the healthy controls had (a history of) neurological disorders.

**Table 1:** Patient and FCD characteristics, and descriptions of the connectivity profiles. Aberrant connectivity profiles (compared to controls) are found in most cases; typically the abnormality in local functional connectivity extended beyond the structural lesion. SF: seizure frequency; low signifies one seizure per month, high signifies multiple seizures per week. IISS: inter ictal seizure spread; low signifies confined to one or several ipsilateral electrodes, high signifies including contralateral electrodes. n.d.: none detected; no epileptiform activity found during EEG acquisition. n.a.: not applicable.

ID	age [y]	gender	SF	IISS	FCD location	Aberrant connectivity profile	Relation to structural lesion
1	30	f	high	high	right insula	hypo	both within and beyond
2	29	f	high	n.d.	right superior frontal	distal transient hypo	only beyond
3	33	m	low	none	right inferior frontal	distal hyper	only beyond
4	47	f	high	none	right precentral	n.a.	n.a.
5	21	m	low	high	left postcentral	distal hyper	both within and beyond
6	47	m	high	low	left posterior cingulate	n.a.	n.a.
7	43	m	high	low	left caudal middle frontal	distal transient hypo	both within and beyond
8	26	m	low	none	left rostral middle frontal	distal transient hypo	only beyond
9	21	f	low	n.d.	caudal anterior cingulate	hyper	both within and beyond
10	21	m	high	none	right supramarginal	proximal hyper, distal hypo	both within and beyond
11	21	m	low	n.d.	right insular	proximal hyper	only within
12	27	m	moderate	none	left precentral	transient hyper	both within and beyond
13	54	m	high	none	right rostral middle frontal	hypo	both within and beyond
14	25	m	none	unknown	left inferior parietal	distal hyper	only beyond

### MR imaging

All subjects underwent structural as well as functional MRI at 3T (Philips Achieva, Best, the Netherlands) using an 8-element receive-only SENSE head-coil.

Structural imaging involved a T1-weighted scan employing the following settings: 3D fast spoiled gradient echo sequence; echo time/repetition time/inversion time (TE/TR/TI) 3.8/8.3/1022 ms; voxel size 1x1x1 mm^3^; and acquisition time 7.5 min. In addition, a fluid attenuated inversion recovery (FLAIR) sequence was used (3D turbo spin echo; TE/TR/TI 330/8000/2400 ms; 0.4x0.4 mm^2^ in plane resolution, 0.6 mm axial slices; and acquisition time 8 min).

Functional MRI involved a blood oxygen level dependent (BOLD) T2*-weighted task-free scan, for which the participants were instructed to close their eyes, lie still, and think of nothing in particular. The settings were: single-shot echo planar imaging (EPI) sequence; TE/TR 35/2000 ms; 2x2 mm^2^ in plane resolution, 4 mm axial slices; 195 dynamics; and acquisition time 6.5 min.

### Neuroradiological examination

The structural scans were reviewed by a board certified neuroradiologist with over 20 years of experience (PH) with the aim to localize the structural lesion. A spherical region of interest was placed at the center of the cortical abnormality on the T1-weighted scan. The radius of this sphere was tuned to comply most optimally with the structural boundaries of the cortical abnormality. All patients had a type II dysplasia [2].

### FCD local functional connectivity

**Fig 2:**
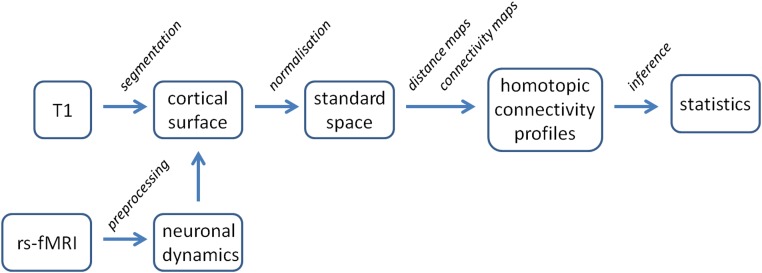
Resting-state fMRI processing pipeline. The cortical surface is segmented from the T1-weighted scan and the preprocessed fMRI data is mapped to the cortex. The gyral pattern is normalized and maps are derived for distance and seed-based functional connectivity to construct profiles of connectivity as a function of distance. These are investigated to find abnormalities in local functional connectivity for the individual patients.

The characterization of (abnormal) local functional connectivity around FCDs required several steps, see Fig 2. An important aspect was the mapping of all native space datasets to a common anatomical standard. This allowed for the derivation of homotopic normative connectivity profiles from the healthy controls to compare the FCD connectivity profiles to. Furthermore, functional connectivity as well as distance maps were to be calculated. It is assumed that locally, functional connectivity is mediated by juxtacortical short association fibers (u-fibers). As a surrogate for the length of these fibers, the distance along the cortical surface (i.e. geodesic distance) was used to construct the required distance maps. Furthermore, functional connectivity maps were derived from preprocessed fMRI data in a seed-based approach. These were combined with the distance maps to calculate connectivity profiles, i.e. connectivity as a function of distance, which were finally used for statistical inference. These steps are summarized in Fig 2 and explained in detail below.

### Registration of FCD locations to anatomical reference space

**Fig 3:**
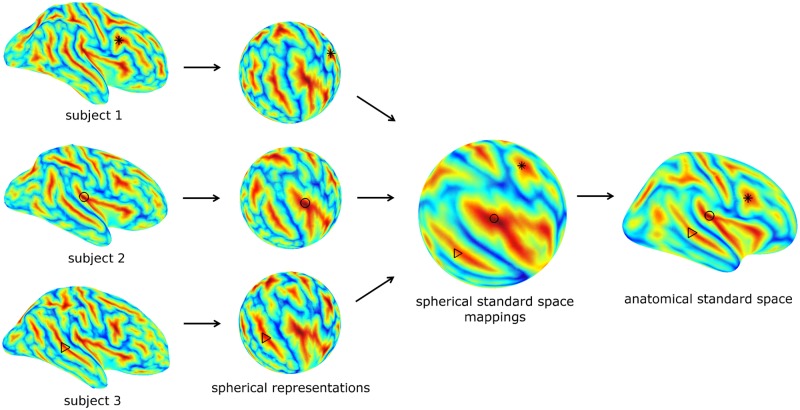
Inflated views of the pial surface of 3 representative subjects; the pattern of gyri/sulci is encoded in blue/red. Alternatively, these gyral patterns may be represented in spherical view. These spherical views can be mapped to a spherical standard space in Freesurfer, which is associated with an anatomical standard space. Data associated with the gyral pattern, such as cortical locations (black markers), are thus mapped to an anatomical standard, which enables homotopic between-subject comparisons.

The Freesurfer software package [16,17] was used to tessellate the cortical surface from the T1-weighted scan; the resulting 3D triangular mesh consisted of approximately 300,000 vertices. For each patient, the dysplasia location was mapped to the nearest vertex (smallest Euclidian distance). Subsequently, the pattern of gyri and sulci was mapped to this cortical surface, and registered with Freesurfer’s spherical standard space, which was subsequently warped to Freesurfer’s standard anatomical space, see Fig 3. Effectively, this procedure maps the individual native space dysplasia locations to a common anatomical reference, suitable for between-subject homotopic comparison. It has been shown that gyral pattern based registration is more robust than conventional voxel-based approaches, and highly accurate compared to manual between-subject region of interest (ROI) placement [18]. For a number of patients, the mappings of the FCD location to several representative controls are illustrated in S1 Fig.

### Geodesic distance

**Fig 4:**
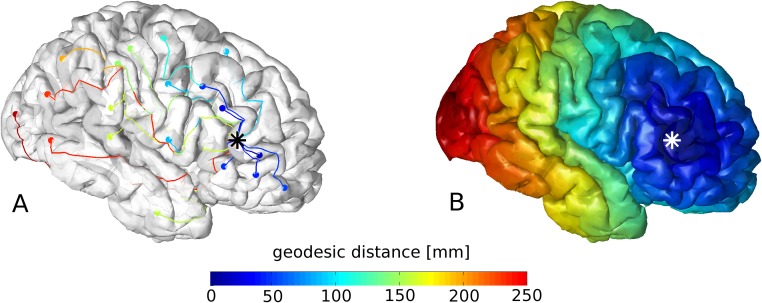
For the cortical location indicated by an asterisk (*), the shortest paths (along the cortex) to a number of other cortex vertices were determined and color coded by their path length in A; combining this for all cortex vertices yields the geodesic distance map in B.

To investigate functional connectivity as a function of distance, the distance maps for the different dysplasia locations were determined in standard space. Assessment of distance maps in standard space compensates for inter-individual difference in the size and relative proportions of gyri and sulci. To construct these distance maps, for each dysplasia vertex, the shortest distance along the cortical surface to each other vertex was calculated, see Fig 4. For this, the exact geodesic algorithm for triangular meshes was used as described by Mitchell et al [19,20] in a Matlab implementation by Kirsanov, see http://code.google.com/p/geodesic/.

### fMRI preprocessing

The preprocessing of the fMRI data was performed using software tools from Statistical Parameteric Mapping (SPM8), FMRIB’s Software Library (FSL 4.1.7) and Freesurfer (version 5.1.0). The two major steps are signal enhancement and projection of the data onto the cortical surface.

### Signal enhancement

First, to correct for head motion, all fMRI volumes were registered to the first dynamic scan using affine registration (6 degrees of freedom). Subsequently, the mean fMRI volume was calculated and used to affinely register the fMRI data to the T1-weighted scan.

Next, the fMRI data were deconfounded using linear regression. As nuisance regressors the movement parameters as derived in the previous step were used, as well as average white matter time series. The aim of this step was to remove global confounds (such as scanner drift and breathing artifacts) from the data, without regressing out any of the neuronal signal fluctuations [21,22]. For this reason, the white matter masks (left and right hemisphere; derived from Freesurfer) were eroded by a single voxel to prevent the mixing in of gray matter signal due to partial volume effects of voxels at the gray matter-white matter interface.

Finally, band pass filtering was applied to confine the signal to the range of 0.01–0.1 Hz typically used in resting-state fMRI analysis [23–25].

### Projecting resting-state data to the cortex

The preprocessed fMRI data was projected onto the cortical surface using Freesurfer tools. This involves averaging the fMRI time-series over the cortex in the direction perpendicular to the surface for each vertex. Methodologically, this is similar to projecting conventional (voxel-based) activation maps to the cortical surface, only here it is part of the analysis pipeline rather than a visualization method.

To avoid artifacts at tissue interfaces (partial volume effects due to the proximity of white matter, CSF, or blood vessels), projections only included the cortical core (80% of the cortical thickness).

Finally, the surface-based data were spatially smoothed (over the surface) using a Gaussian kernel of full-width-at-half-maximum (FWHM) 10 mm.

### Extraction of seed time series

For each patient, a seed time series was calculated by averaging the time series over all vertices within 5 mm geodesic distance of the dysplasia location. Using the same approach, homotopic time series were derived in all other subjects.

It is known that epileptiform spikes may induce additional BOLD signal fluctuations [26]. Therefore, patient and controls seed time series were compared to detect increased signal variance in FCD time series using 2-sample Student’s t-tests (p<0.05). The goal of this step was to exclude patients with spike-contaminated seed time series from further analysis, since these may perturb the connectivity profiles as described below.

### Derivation and comparison of local functional connectivity profiles

For each patient, the seed time series was correlated with those of all other vertices. The resulting connectivity map was combined with the associated geodesic distance map to assess functional connectivity as a function of distance: for concentric isobands on the distance map of width 2 mm, the average connectivity was calculated for the local environment in the range 2–40 mm. Similarly, homotopic connectivity profiles were derived in all other subjects.

### Statistical analysis

Two-sample Student’s t-tests were used to compare the FCD connectivity profile of each individual patient to the set of homotopic normative connectivity profiles derived from the healthy control subjects. To investigate the robustness of the approach, two-sample Student’s t-tests were additionally employed to assess whether the homotopic (i.e. the anatomically comparable regions) connectivity profiles of (non-lesional) control regions in the other patients, who had their dysplasia in a different location, were abnormal with respect to the normative connectivity profiles derived from he healthy control subjects.

Connectivity abnormalities were considered relevant if deviations from the normative profiles were both significant (p<0.01) and observed over multiple consecutive isobands of distance.

## Results

### Major findings

In a single patient, the FCD time series showed increased signal variance (p = 0.01); the remaining 14 patients were taken forward to further analysis.

In the healthy controls, gradually and consistently decreasing connectivity profiles were found, the course of which varied with cortical location. In the majority of patients (12/14), the connectivity profile of their FCD was significantly disturbed with respect to the corresponding homotopic normative profiles of derived from the controls. For 8/14 patients, connectivity was already disturbed within the FCD. These results are summarized in Table 1.

**Fig 5:**
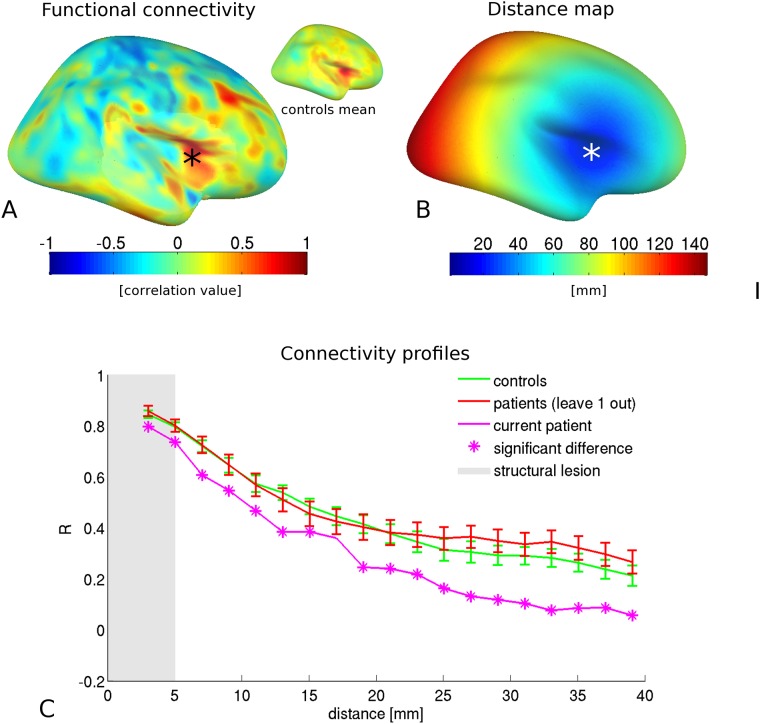
For one exemplary patient (patient 1), the lesion location and its connectivity map are given in A; the inset is the average homotopic connectivity map of the controls. The geodesic distance map for this location is given in B. By combining the connectivity and distance maps, connectivity profiles as depicted in C can be constructed. Also average homotopic connectivity profiles for the healthy control subjects and for the other patients (i.e. excluding patient 1) are provided in C, showing no significant differences in connectivity profiles between the healthy controls and the other patients (without lesions at the lesion location of patient 1). The extent of the structural lesion on conventional MRI is shaded; connectivity values of the single patient that are aberrant compared to the set of normative control curves are indicated by an asterisk (*). Surface maps represent inflated views; in contrast, connectivity values and distance maps were assessed in native space. Error bars represent 1 standard error.

Homotopic connectivity profiles derived from other patients, beyond their own FCD, demonstrated no deviations from the normative curves, obtained from healthy control subjects, at the group level, see Fig 5C. Aberrant FCD connectivity profiles both involved local hyper- and hypoconnectivity, as well as combinations; for an overview, see Table 1. Specific illustrative cases are discussed below.

### Overall hypoconnectivity

For the same patient as in Fig 1 (patient 1; right insular FCD), a profile of overall hypoconnectivity was found, see Fig 5. Note that already within the structural lesion, functional connectivity was reduced with respect to the homotopic connectivity profiles derived in the controls. This patient had a high seizure frequency (multiple seizures per week) and high inter-ictal spread of epileptiform activity (i.e. including propagation to contralateral electrode).

### Transient hypoconnectivity

**Fig 6:**
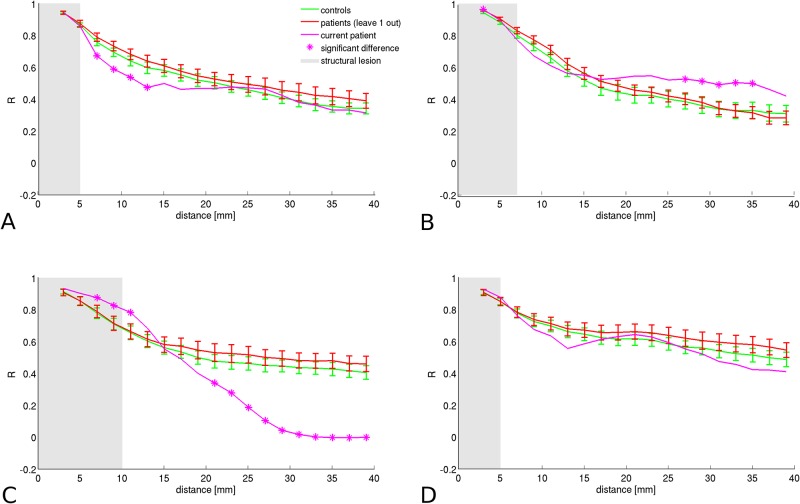
Different profiles of aberrant connectivity. In patient 2, transient hypoconnectivity is found directly beyond the lesion visible on conventional structural MRI (A). In patient 5, on the other hand, transient distal hyperconnectivity is found (B). In patient 10, a dual profile is found of hyperconnectivity within and directly beyond the structural lesion, and hypoconnectivity more distally (C). In a few patients, no aberrant connectivity values were found, such as in patient 4 (D). Error bars represent 1 standard error.

A similar but more subtle profile of aberrant connectivity was found in patient 2, see Fig 6A. This patient had a right superior frontal lesion and displayed reduced functional connectivity directly adjacent to the structural lesion, up to a distance of about 12 mm from the lesion center. This patient had a high seizure frequency. No statement can be made about interictal spread of epileptiform spikes since none were detected during EEG acquisition.

### Distal hyperconnectivity

Also hyperconnectivity was found, for example in patient 5, who had a left postcentral lesion, see Fig 6B. Local connectivity was normal up to a distance of about 25 mm, i.e. well outside the boundary of the structural lesion. Beyond this distance, hyperconnectivity was found for about 10 mm. This patient had a low seizure frequency (about 1 seizure per month) combined with high interictal spread of epileptiform activity.

### Proximal hyperconnectivity, distal hypoconnectivity

In patient 10, who had an FCD in the right supramarginal gyrus, connectivity was significantly increased with respect to the control curves up until 10 mm from the lesion center. It then transitioned into a profile of significant hypoconnectivity beyond the structural lesion, see Fig 6C. This patient had a high seizure frequency and no interictal spread of epileptiform activity.

### No abnormalities

In patient 4, who had a right precentral FCD, no abnormalities were found in the connectivity profile, see Fig 6D. This patient combined a high seizure frequency with low interictal spread of epileptiform EEG-activity (i.e. confined to one or several ipsilateral electrodes).

Overall, note that no aberrant connectivity profiles were found in the patients beyond their FCD location. This was assessed using group-wise comparison of the leave-one-out patient curves to the control curves.

## Discussion

In this study, we explored local functional connectivity of FCD lesions. Since these cortical malformations vary in location over patients, dedicated methodology for individual subject analysis was developed. This involved gyral pattern registration of the dysplasia locations as well as the fMRI data to a common anatomical standard, for comparison of individual patient connectivity profiles to the corresponding homotopic normative profiles derived in healthy controls.

### Major findings

Local connectivity profiles were abnormal in the majority of patients (12/14). Remarkably, these aberrant connectivity profiles represented hyperconnectivity, hypoconnectivity and combinations thereof. Typically, functional connectivity was (also) aberrant beyond the structural boundaries of the lesion (11/14 cases). Furthermore, patient connectivity profiles were normal at distal locations (i.e. at the FCD location of *other* patients).

### Pathophysiological interpretation

The variable nature of the abnormalities in functional connectivity may suggest that different pathological mechanisms are at work, such as excitotoxic effects or the formation of local epileptic networks, causing hypo- and hyperconnectivity, respectively. The latter has been suggested as a mechanism to recruit non-dysplastic cortex in FCD seizure generation during sleep [14]. These variable findings may reflect the heterogeneity of a clinical sample of FCD patients, with variations in frequency of interictal epileptiform discharges (IEDs) or anti-epileptic drug (AED) use. Future studies may consider a more homogeneous FCD cohort. Since we assess functional connectivity employing correlation of BOLD time series, our findings may alternatively reflect localized dysfunction of neurovascular coupling. Future studies employing larger subject numbers are called for to verify the reproducibility of these variable findings, and should aim to shed light on the underlying pathophysiology.

With respect to the extent of the functional connectivity abnormality, typically connectivity profiles were aberrant also beyond the MR-visible lesion (11/14), which is in line with histopathological findings [3]. Further research is needed to establish whether the current findings translate to specific local microscopic tissue abnormalities, e.g. combining presurgical assessment of functional connectivity with postsurgical histopathological examination of resection specimens. It would also be of interest to study whether patients with residual areas of abnormal local connectivity have a higher incidence of postsurgical seizures. This would post-hoc confirm the added value of assessing local functional connectivity abnormalities for delineating the surgically relevant lesion.

### Clinical outlook

In this methodological and exploratory study, no clear links were found between the type of aberrant connectivity profile and patient characteristics such as EEG findings, see Table 1. However, functional imaging has been linked to electrophysiology before, in studies were epileptiform spikes were mapped to the brain using simultaneously acquired EEG-fMRI [27,28]. Possibly more advanced EEG data, such as recorded from subdural electro grids, may help to relate the current functional connectivity findings to subject-specific electrophysiology. In the current clinical cohort, in which EEG data was acquired as part of the diagnostic work-up, nu such detailed electrophysiological information was available.

We also found that at the group level, patient connectivity profiles are normal beyond their dysplasia location (i.e. at the locations were *another* patient had a dysplasia). This suggests that in FCD-mediated epilepsy, functional connectivity is not globally disturbed throughout the brain, but to some extent specifically related to the FCD lesion. In line with this, FCD-related seizures are typically stereotyped, suggesting the involvement of well-localized brain regions [14]. This may also explain why focal resection may have such excellent seizure control outcome [8].

### Methodological considerations

We investigated functional connectivity up to a range of 40 mm geodesic distance from the dysplasia center. This corresponds roughly to the extent of a gyrus, and resulted (for the controls) in gradually and consistently decreasing connectivity profiles, as expected. In the majority of the investigated patients, functional connectivity was disturbed right up to the end of this range, and beyond the border of the structural lesion. This may indicate abnormal distal connectivity; however, the investigation of connectivity between distributed regions as mediated by long-range connections is beyond the scope of this study.

Connectivity profiles (as a function of distance) were assessed for concentric isobands on the distance map of width 2 mm in this study of methodological development. The optimization of these and other parameters is an important subject for future research.

It is remarked that the definition of the border of the structural lesion is not very accurate as the representations of an FCD by a spherical region of interest is a strong simplification. In any case, our findings suggest that in FCD the abnormality extends beyond the MR-visible lesion. As such, our findings might be of relevance in surgical planning, as recent findings show that the identification and removal of as much aberrant cortex as possible improves seizure-free outcome [3,8].

For 8/14 patients, functional connectivity was already aberrant within the lesion, whereas in others the connectivity profile only became aberrant beyond the structural margins. Given what is said about the accuracy of the extent of the structural lesion, these differences may also arise from the definition of the seed time series, which was derived from all vertices within 5 mm (geodesic distance) from the lesion midpoint. At short distance and in large lesions, this comes down to assessing the (high) connectivity of the core of the lesion with its outer perimeter, whereas in small lesions, the (lower) connectivity with the surrounding tissue was calculated. We used variance analysis of the seed time series in an attempt to prevent connectivity profile differences to be driven by seed time series abnormalities.

In the current approach we statistically compared single patients with a healthy control group and the other patients in which the single patient was left out. Patient and control groups were, however limited in size (<20), which may provide uncertainties in the variances of the populations. Another approach could be the application of bootstrapping in which samples were repeatedly replaced.

### Directional anisotropy of effects

In our approach we averaged functional connectivity values over concentric isobands on the geodesic distance map. With increasing distance from the dysplasia center, this lumps more and more distributed regions into a single connectivity value, gradually compromising the spatial precision of our approach. However, our work represents a study aimed at investigating homogenous local (rather than distributed distal) connectivity profiles in symptomatic epilepsy. The exact extent of the local environment, preferably as a function of the location on the cortex and taking into account local gyration, is an interesting and challenging subject for future research. Since the cortex is organized into a mosaic of functionally specialized regions (segregation) that interact (integration), such approaches should confine the local analysis to e.g. single gyri in order not to cross functional boundaries [29].

## Conclusion

Methodology was developed that shows that functional connectivity is disturbed in FCD, which may express as hyperconnectivity, hypoconnectivity or combinations. The histopathological and electrophysiological bases of these findings remain to be established. It was demonstrated that in FCD that local functional connectivity profiles reveal abnormalities beyond the structural lesion, while functional connectivity profiles that are not related to a lesion appear normal. In the future, the methodology we introduced here in a ‘proof of principle’ analysis that has potential to facilitate the delineation of the clinically relevant lesion beyond its structural boundaries (as derived from conventional MRI), and may thus improve post-surgical outcome. The developed approach and obtained results require further cross-validation by independent patient groups and electrophysiological and histopathological validation in future studies.

## Supporting Information

S1 Fig For patients 1–3, the mappings of the lesions to the homotopic locations in controls 1–3 are given. These registration are of a global nature and ensure high accuracy, also in the case of subtle local gyral abnormalities. Inflated views of the pial surface are visualized, the pattern of gyri/sulci is encoded in blue/red.
(TIFF)
