# Supplementary figures and images for: Abnormal Profiles of Local Functional Connectivity Proximal to Focal Cortical Dysplasias
Source: PLoS One. 2016 Nov 18;11(11):e0166022. doi: 10.1371/journal.pone.0166022 (PMC5115673; doi:10.1371/journal.pone.0166022)

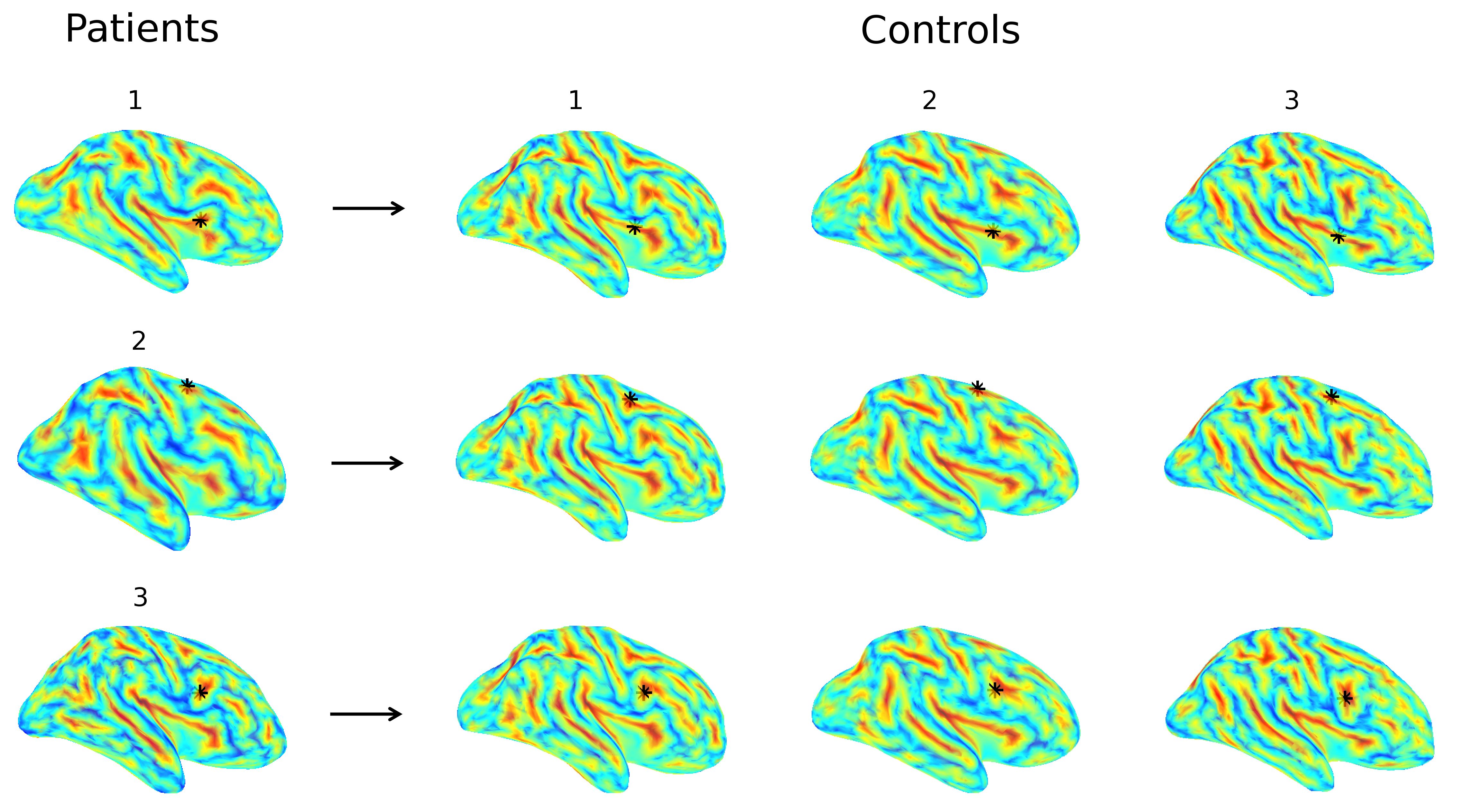

Supplement: S1 Fig — These registration are of a global nature and ensure high accuracy, also in the case of subtle local gyral abnormalities. Inflated views of the pial surface are visualized, the pattern of gyri/sulci is encoded in blue/red. (TIFF) [file pone.0166022.s001.tiff]
